# Competitive and cooperative arm rehabilitation games played by a patient and unimpaired person: effects on motivation and exercise intensity

**DOI:** 10.1186/s12984-017-0231-4

**Published:** 2017-03-23

**Authors:** Maja Goršič, Imre Cikajlo, Domen Novak

**Affiliations:** 1 Department of Electrical and Computer Engineering, University of Wyoming, 1000 E University Avenue, Laramie, WY 82071 USA; 2 University Rehabilitation Institute, Republic of Slovenia, Linhartova 51, SI 1000 Ljubljana, Slovenia

**Keywords:** Rehabilitation, Virtual reality, Multiplayer games, Interpersonal rehabilitation games, Social interaction, Motivation, Exercise intensity

## Abstract

**Background:**

People with chronic arm impairment should exercise intensely to regain their abilities, but frequently lack motivation, leading to poor rehabilitation outcome. One promising way to increase motivation is through interpersonal rehabilitation games, which allow patients to compete or cooperate together with other people. However, such games have mainly been evaluated with unimpaired subjects, and little is known about how they affect motivation and exercise intensity in people with chronic arm impairment.

**Methods:**

We designed four different arm rehabilitation games that are played by a person with arm impairment and their unimpaired friend, relative or occupational therapist. One is a competitive game (both people compete against each other), two are cooperative games (both people work together against the computer) and one is a single-player game (played only by the impaired person against the computer). The games were played by 29 participants with chronic arm impairment, of which 19 were accompanied by their friend or relative and 10 were accompanied by their occupational therapist. Each participant played all four games within a single session. Participants’ subjective experience was quantified using the Intrinsic Motivation Inventory questionnaire after each game, as well as a final questionnaire about game preferences. Their exercise intensity was quantified using wearable inertial sensors that measured hand velocity in each game.

**Results:**

Of the 29 impaired participants, 12 chose the competitive game as their favorite, 12 chose a cooperative game, and 5 preferred to exercise alone. Participants who chose the competitive game as their favorite showed increased motivation and exercise intensity in that game compared to other games. Participants who chose a cooperative game as their favorite also showed increased motivation in cooperative games, but not increased exercise intensity.

**Conclusions:**

Since both motivation and intensity are positively correlated with rehabilitation outcome, competitive games have high potential to lead to functional improvement and increased quality of life for patients compared to conventional rehabilitation exercises. Cooperative games do not increase exercise intensity, but could still increase motivation of patients who do not enjoy competition. However, such games need to be tested in longer, multisession studies to determine whether the observed increases in motivation and exercise intensity persist over a longer period of time and whether they positively affect rehabilitation outcome.

**Trial registration:**

The study is not a clinical trial. While human subjects are involved, they participate in a single-session evaluation of a rehabilitation game rather than a full rehabilitation intervention, and no health outcomes are examined.

**Electronic supplementary material:**

The online version of this article (doi:10.1186/s12984-017-0231-4) contains supplementary material, which is available to authorized users.

## Background

### Home rehabilitation technology

Diseases such as stroke have a massively debilitating effect on people’s lives. It is estimated that one in six people will experience a stroke in their lifetime [1], and 88% of survivors report some impairment of their limb function [2]. In the United States, approximately 795,000 individuals suffer a new or recurrent stroke every year, leading to an estimated combined direct and indirect cost of $68.9 billion [3]. Intensive training delivered by a therapist soon after the injury can effectively restore motor functions needed for independent life. However, even top hospitals only devote a limited amount of time to rehabilitation of motor functions [4]. The situation is even worse in most other hospitals and health centers, where patients are idle for most of the day due to a shortage of qualified medical staff [4]. After leaving the hospital, patients thus need to exercise at home without therapist supervision in order to fully regain their abilities.

Several technologies, ranging from consumer devices such as the Microsoft Kinect [5] to complex exoskeletons [6], have been deployed for motor rehabilitation at home. These technologies usually combine limb tracking with virtual environments presented on a personal computer, which allow patients to perform a variety of simulated activities of daily living [7]. Furthermore, they incorporate game-like elements such as automated difficulty adaptation, score displays and cognitive challenges [8–11]. However, despite promising technical achievements, the effectiveness of home rehabilitation technology remains limited. A recent study showed that, even if a therapist prescribes a technology-supported exercise, only about 30% of unsupervised patients will comply with the rehabilitation regimen [12].

This lack of compliance is due to lack of motivation for rehabilitation, which is known to be a key determinant of rehabilitation outcome: patients who are unmotivated will not exercise frequently or intensely enough [13, 14]. Studies outside rehabilitation have already shown that motivational interventions improve compliance with the therapy regimen [15], and recent home rehabilitation studies have emphasized the importance of motivational elements that would increase the duration and intensity of exercise [16, 17].

### Interpersonal rehabilitation games

One possible way to improve motivation in motor rehabilitation is through the use of interpersonal rehabilitation games, which allow the patient to compete or cooperate with another person. Competition and cooperation are known to be very motivating in games for entertainment [18, 19], but also have potential benefits for health. For example, cooperative games are known to increase motivation and energy expenditure in weight loss regimens [20].

Interpersonal rehabilitation games were first proposed in 2006 and envisioned as cooperation between a patient and therapist [21, 22], though competition between two patients was proposed soon afterwards [23, 24]. A first evaluation found that unimpaired subjects prefer playing rehabilitation games against a fellow human than against a computer, though the evaluation did not involve patients [24]. Similarly, a pilot study found that stroke survivors prefer a two-player version of a rehabilitation game to a single-player version and exhibit larger arm movements in the two-player version [25].

Designing interpersonal rehabilitation games, however, is not trivial, as not all patients will enjoy competing or cooperating with other people. In our previous work, we developed an arm rehabilitation game (air hockey) that can be played either against a computer opponent, against another human player, or together with another human player against the computer [26]. All three variants were tested in single sessions with 30 unimpaired subjects and 8 stroke survivors. We found that both competition and cooperation resulted in higher enjoyment than exercising alone, but that participants who enjoyed competition generally did not enjoy cooperation and vice versa. These preferences could be predicted from personality: extraverted, competitive participants preferred competition while introverted, emotionally unstable and uncompetitive participants preferred cooperation. Furthermore, pairs of friends had an overall more enjoyable experience than pairs of strangers or acquaintances.

### Interpersonal games for home rehabilitation

Home rehabilitation presents a different situation than clinical rehabilitation: a therapist is rarely available, and we cannot expect patients (who are often unable to travel independently) to regularly meet with other patients, particularly in rural areas. However, patients could still play interpersonal rehabilitation games together with their friends or relatives. Competition and cooperation between a patient and their friend or relative were previously suggested (though not tested) by Alankus et al. [11] and Vanacken et al. [27], who felt that this would increase patient motivation by strengthening social bonds. This possibility is supported by our previous study [26], which showed that people who know and like each other are likely to have a more enjoyable experience in interpersonal rehabilitation games.

Alankus et al. [11] suggested that cooperation would likely be a better option for games played by a patient and their family member, as unimpaired people may find the notion of competing against someone who is struggling to control their arm to be uncomfortable. Nonetheless, a recent study performed the first evaluation of a competitive rehabilitation game played by two stroke patients together with their spouses [28]. Both the patients and spouses enjoyed the game, suggesting that competition may still have a place in motor rehabilitation at home. Cooperation between a patient and their friend or relative, on the other hand, has not been tested in motor rehabilitation, and its benefits remain purely hypothetical.

The objectives of the current study are to evaluate the short-term effects of competitive and cooperative arm rehabilitation games played by a patient and an unimpaired person together. While the main application for such games is home rehabilitation (patient and friend/relative), they could in principle also be used in clinical settings (patient and therapist). Therefore, we recruited a total of 29 patients with arm impairment, of which 19 played multiple variants (playing alone, competition, cooperation) of an arm rehabilitation game with a friend/relative and 10 played with an occupational therapist. All patients participated in a single session, and our research questions were:

- **Q1**: Does competing or cooperating with an unimpaired person result in higher motivation than exercising alone?
- **Q2**: Does competing or cooperating with an unimpaired person result in higher exercise intensity than exercising alone?
- **Q3**: How do potential benefits of competition and cooperation depend on the patient’s personality and the type of unimpaired co-player (friend/relative or therapist)?

A preliminary version of this work was presented at the EMBC 2016 conference [29]. The conference version includes information about hardware and software as well as limited data analysis for the first 7 impaired participants.

## Methods

### Participants

A total of 30 people with arm impairment were recruited for the study. One was deemed unable to give informed consent and did not undergo the study protocol, resulting in 29 valid participants (15 male, 14 female). They were 56.7 ± 14.7 years old (mean ± SD). Their arm impairment was due to either ischemic stroke (19 participants), hemorrhagic stroke (3 participants), brain tumor surgery (4 participants), shoulder rotator cuff tear (2 participants) or traumatic brain injury (1 participant). The degree of arm impairment was tested with the Box and Block test of manual dexterity [30], which yielded a score of 32.9 ± 21.1 points. The Box and Block tests a person’s ability to perform reaching and grasping motions, with a score of zero indicating no motion ability and scores above approximately 60 indicating normal (unimpaired) motion ability.

The impaired participants were divided into two groups: 19 participated in a session together with their friend or relative while 10 participated together with their occupational therapist. The two groups differed as follows:

- Participants who were joined by their friend or relative had already completed their clinical rehabilitation program. Time since injury was 9.5 ± 5.8 years, minimum 6 months, maximum 18 years. Their Box and Block score was 30.2 ± 23.3 points. Ten had an impaired left arm and nine had an impaired right arm. The experiment session was conducted either at their home or at a community center with which they were familiar. They were recruited among the population of the US states of Wyoming and Colorado as well as the Republic of Slovenia. Nine participants were joined by a friend, nine were joined by their spouse, and one was joined by her adult son.
- Participants who were joined by their occupational therapist were actively engaged in their clinical rehabilitation program at the University Rehabilitation Institute of the Republic of Slovenia. Time since injury was 5.5 ± 5.0 months, minimum 2 months, maximum 18 months. Their Box and Block score was 37.9 ± 15.9 points. Four had an impaired left arm and six had an impaired right arm. The experiment session was conducted in the occupational therapy section of the University Rehabilitation Institute, and the same therapist participated in all sessions.

Participants had no cognitive impairments that would prevent them from understanding the experiment, providing informed consent or following the experimenter’s instructions. All sessions were conducted in late 2015 and early 2016.

### Hardware and software

#### Rehabilitation device

**Fig. 1:**
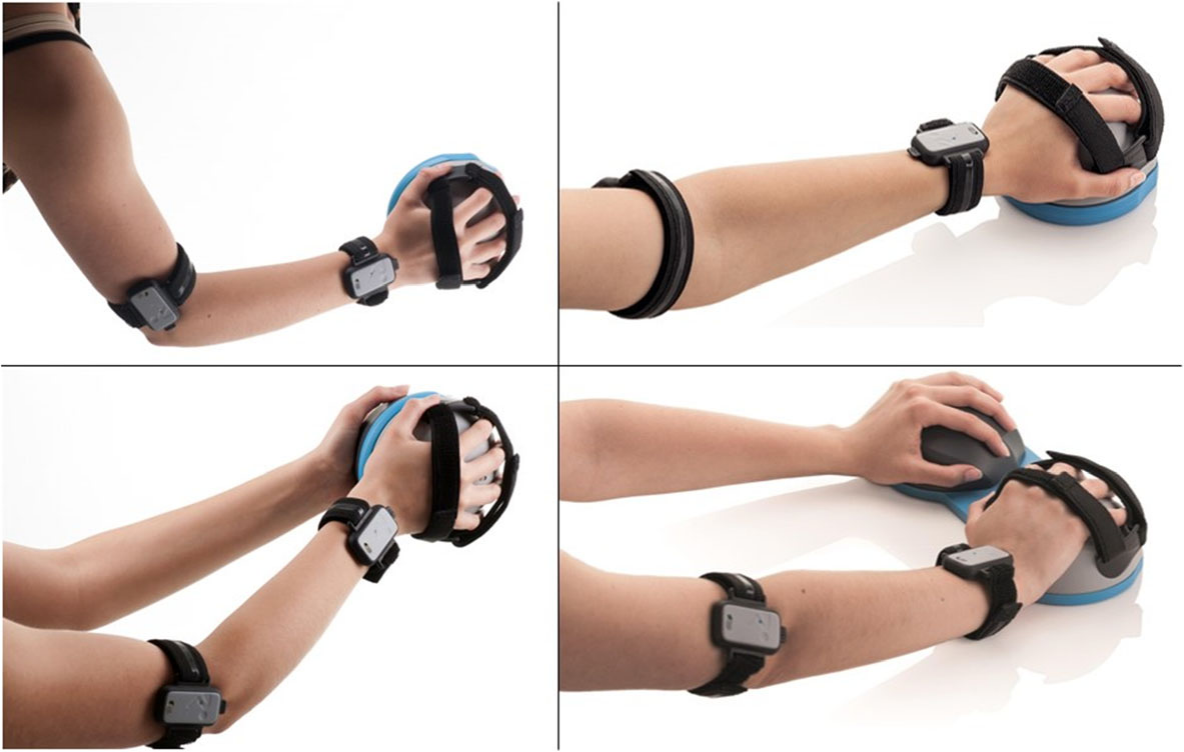
The BiMeo used unimanually without support (*top left*), unimanually on a table (*top right*), bimanually without support (*bottom left*), and bimanually on a table (*bottom right*)

The Bimeo (Kinestica d.o.o, Slovenia) is a commercially available arm rehabilitation system that consists of two inertial measurement units (IMUs) on the upper arm and forearm as well as a handle that contains a third IMU and a force sensor (Fig. 1). Each IMU consists of an accelerometer, gyroscope and magnetometer. Sensor fusion algorithms are applied to the raw outputs of each sensor to calculate the orientation of each IMU, joint angles, and hand position in three-dimensional space. Previous work with these IMUs has shown that they can track joint angles with an accuracy of approximately 2° in normal conditions and 5° in worst-case conditions [31, 32]. The position of the hand (end-effector) can also be tracked with an accuracy of ±1 cm for each horizontal dimension in normal conditions.

The BiMeo can be used in different unimanual or bimanual configurations depending on the patient’s motor abilities (Fig. 1). In the unimanual configuration, patients can perform three-dimensional motions with no support (Fig. 1, top left) or two-dimensional motions on a flat surface (Fig. 1, top right). In the bimanual configuration, patients hold the handle with both hands, supporting the motion of the impaired limb with the unimpaired limb (Fig. 1, bottom left). In the case of severe impairment, bimanual motions can be done on a flat surface using a special handle (Fig. 1, bottom right). The force sensor in the handle allows the interaction force between hands to be measured.

All participants controlled the BiMeo with their impaired arm. Since they had different levels of motor impairment, the BiMeo configuration was selected accordingly. Eighteen impaired participants performed left-right unimanual motions without support (Fig. 1, top left), 7 performed unimanual motions on a table (Fig. 1, top right), 1 performed bimanual motions without support (Fig. 1, bottom left), and 3 performed bimanual motions on a table (Fig. 1, bottom right). All unimpaired participants used a Logitech joystick, which was tilted left or right to control the rehabilitation games.

#### Rehabilitation games

**Fig. 2:**
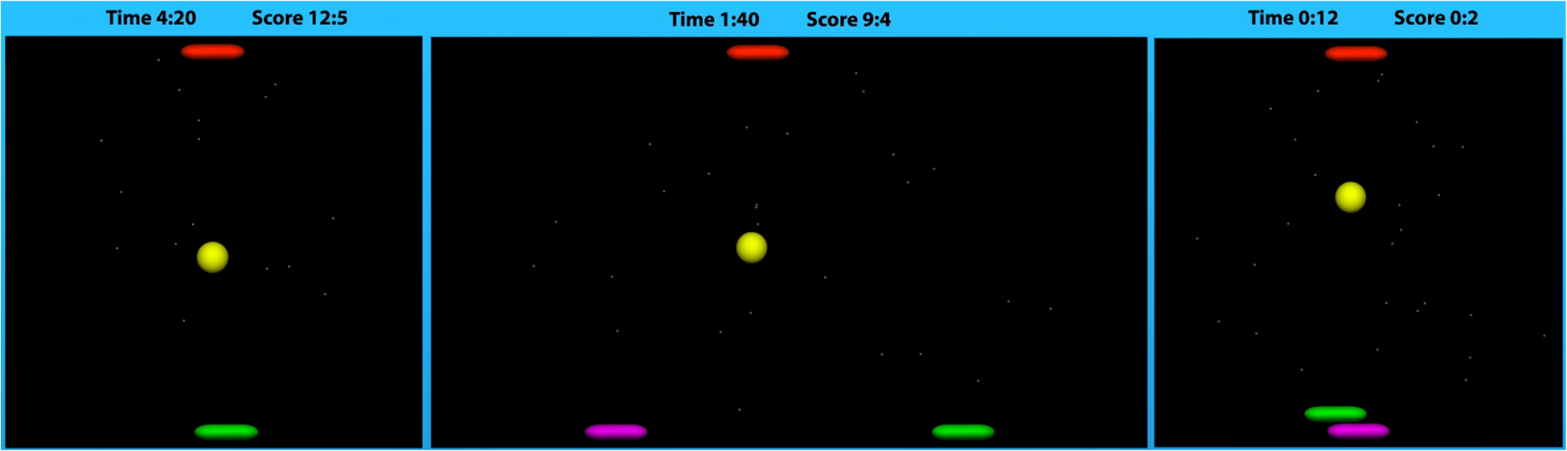
The rehabilitation games. From left to right: single-player / competitive game (which look the same), cooperative game with split field, cooperative game with shared field

We implemented four rehabilitation games that are all variants of the classic game of Pong (Fig. 2). Three games are similar to those used in our previous work [26] while the fourth (cooperative with shared field) was developed specifically for this study. The games are:

- Single-player game: The playing field consists of one paddle at the bottom of the screen, one paddle at the top of the screen, and a ball bouncing between them. The impaired participant moves the bottom paddle by moving the arm left and right. The top paddle is controlled by the computer. The participant’s goal is to keep the ball from reaching the bottom of the screen by intercepting it with the paddle. If the participant intercepts the ball, it bounces off the paddle and moves toward the other side of the screen. Similarly, the computer’s goal is to keep the ball from reaching the top of the screen. Each time the ball hits the top/bottom of the screen, the opposing side scores a point. The ball then reappears at the center of the screen and begins moving in a random direction.
- Competitive game: The game looks the same as the single-player game. The only difference is that the paddle at the top of the screen is now controlled by the unimpaired participant, who moves it by tilting the joystick left and right. The two participants thus compete against each other.
- Cooperative with split field: The game area is twice as wide as in the first two games. Each participant controls a paddle near the bottom of the screen: the impaired participant on the right and the unimpaired participant on the left. However, each paddle can only move half the width of the screen (i.e. from the edge to the center). There is a single paddle near the top of the screen, controlled by the computer. Both participants play together against the computer; however, since each paddle is limited to half the width of the screen, they cannot help each other.
- Cooperative with shared field: The game looks similar to the single-player version. Both the impaired and unimpaired participant control paddles at the bottom of the screen, with the unimpaired participant’s paddle below the impaired participant’s. Both participants again play together against the computer-controlled paddle, with the unimpaired participant paddle acting as “back-up” for the impaired participant.

The speed of the ball was the same for all participants and in all games. It was set based on pilot trials of the games and provided a moderate challenge for all participants.

### Study protocol

At the start of the experiment, both participants were informed about the study purpose and procedure, then signed an informed consent form. They were seated in front of the computer screen, and the BiMeo was attached to the impaired arm of the impaired participant. A simple calibration procedure was performed and the required BiMeo range of motion was adjusted to the impaired participant’s capabilities. The default range of motion was 40 cm from left to right, and was lowered to a minimum of 30 cm for our most severely impaired participants. Participants then practiced playing each game for 30–60 s.

**Fig. 3:**
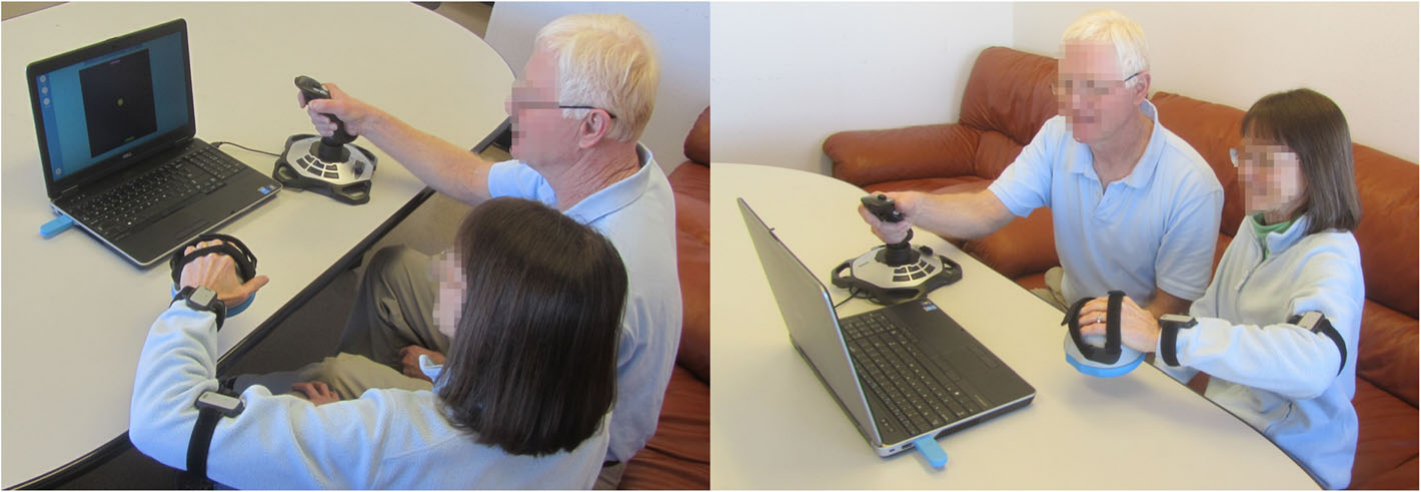
Experimental setup. An impaired participant (left) wears the BiMeo device and exercises together with an unimpaired participant (in this case, their spouse), who uses a joystick. The games are displayed on the laptop in front of them

After practice, all four games were played in random order for 3 min each. This experimental setup is shown in Fig. 3. After each game, impaired participants and unimpaired friends/relatives filled out a questionnaire about that game (see next section). After playing all four games, participants also filled out an overall experience questionnaire and an optional personality questionnaire. The impaired participant completed the Box and Block test of manual dexterity.

### Questionnaires

#### Experience with each game

Impaired and unimpaired participants’ subjective experiences with each game were evaluated using the Intrinsic Motivation Inventory (IMI) [33], which was presented immediately after the 3-min game. The IMI has been previously used with virtual environments for motor rehabilitation [8, 9, 26] and measures four scales: interest/enjoyment, perceived competence, effort/importance, and pressure/tension. While different versions of the IMI exist, the one we used was identical to the one in our previous studies [9, 26] and consists of 20 statements (5 per scale). Participants rate how true each statement is on a 7-point scale, with 1 indicating “not at all true” and 7 indicating “very true”. The possible range for each scale is therefore 5–35.

Participants were asked to rate each statement only as it applies to that game. When rating each game, they were able to see their ratings for all previous games (for example, when rating the third game, they could see their ratings for the first and second games). While somewhat controversial, this approach was tested in our previous work [26] and found to better emphasize differences between games. A full copy of the IMI is available in our previous paper [26].

#### Overall experience questionnaire

After finishing all four games, participants were given a questionnaire that asked them to compare the four games. It consisted of eight questions:

- “What was your favorite game? Why?”
- “What was your least favorite game?”
- “Which game did you put the most effort into?”
- “Which game did you put the least effort into?”
- “Which game did you feel the most competent at?”
- “Which game did you feel the least competent at?”
- “Which game was the most stressful?”
- “Which game was the least stressful?”

The questionnaire focuses on the same four aspects of subjective experience as the IMI, but explicitly asks participants to compare the four games. It was introduced in our previous work [26] since the IMI was found to be poor at identifying differences between similar games.

#### Personality

Participants’ personalities were primarily assessed using the Big Five factor markers: extraversion, agreeableness, conscientiousness, emotional stability, and intellect/imagination. These factors have been used to analyze the effects of personality on game enjoyment in a number of studies [34, 35]. They were measured using the 50-item International Personality Item Pool (IPIP) [36] available at http://ipip.ori.org.

Additionally, the Revised Competitiveness Index [37] was used to measure the subject’s preference for competitive situations. The index consists of two factors, Enjoyment of Competition and Conscientiousness. Only the Enjoyment of Competition factor (hereafter referred to as ‘competitiveness’) was used in our study, as conscientiousness is already measured by the IPIP.

The items of both the IPIP and the Revised Competitiveness Index are brief statements that the subject can agree or disagree with on a 5-point scale from 1 (completely disagree) to 5 (completely agree). All 59 statements (10 for each of the five IPIP factors, 9 for Enjoyment of Competition) were mixed together in a random order. The possible range is therefore 9–45 for competitiveness and 10–50 for the other scales. A full copy of the personality questionnaire is available in our previous paper [26].

The personality questionnaire was presented only to the 19 participants who played together with a friend or relative; the 10 participants who played together with the therapist did not complete it. We acknowledge that it would have been preferable to have all 29 impaired participants complete the personality questionnaire, and it was omitted for logistical and administrative reasons rather than scientific ones.

### Measurement of exercise intensity

**Fig. 4:**
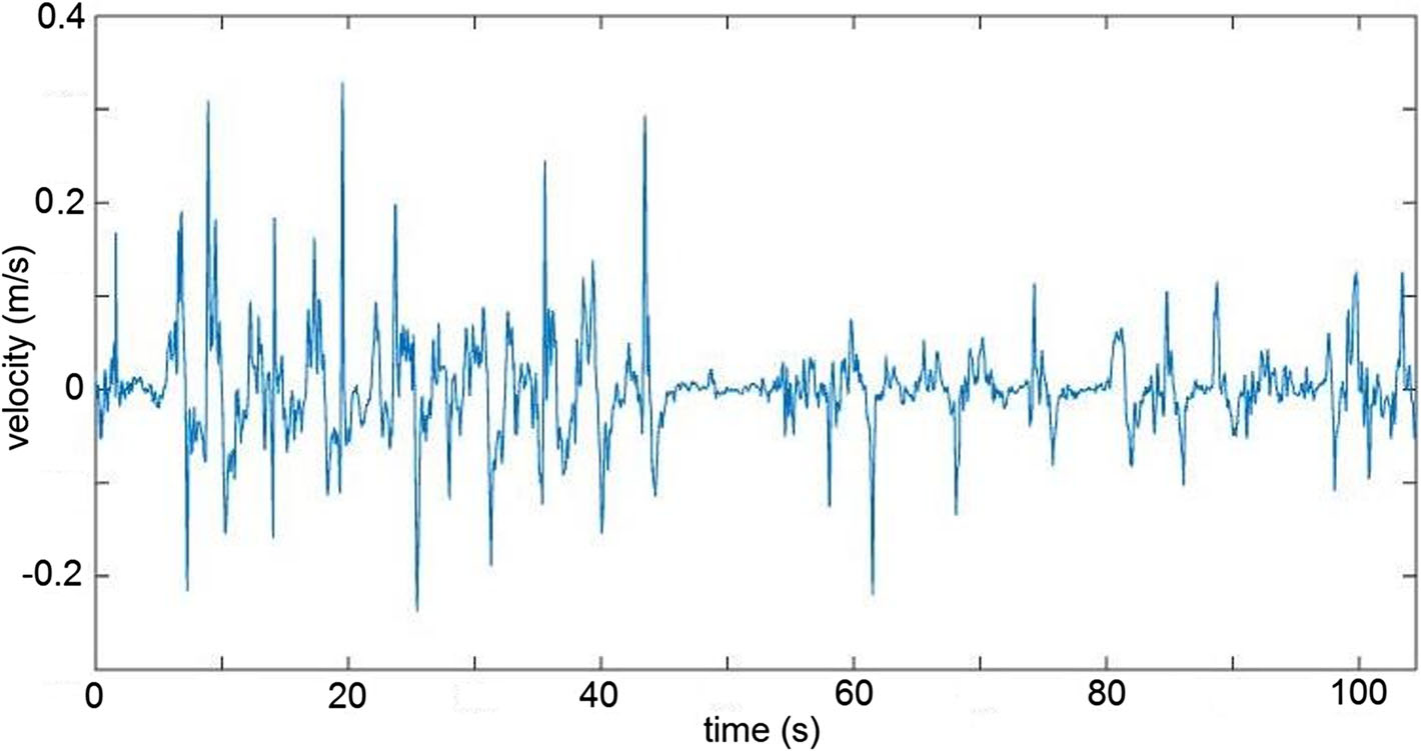
An impaired participant’s hand velocity in the single-player game. The first 50 s are a high-intensity exercise interval while the second 50 s are a low-intensity interval

The hand position was logged by the BiMeo throughout all four games. From the position, the impaired participant’s hand velocity was calculated in the horizontal left-right direction (which is used to control the game). An example of the hand velocity signal is shown in Fig. 4. The root-mean-square (RMS) value and mean absolute value of hand velocity were calculated for each 3-min game interval. The RMS value of hand velocity is known to be an adequate approximation of energy expenditure during upper limb exercise in comparison to measurements derived from the heart’s electrical activity, muscle activation and oxygen consumption [38, 39] and thus serves as the primary measure of exercise intensity. The mean absolute value was calculated as an additional variable that may emphasize different types of motions – compared to the mean absolute, the RMS value is more likely to emphasize very fast, jerky motions.

### Data analysis

#### Subjective experience – Impaired participants

The overall experience questionnaire was analyzed using descriptive statistics to determine how many participants gave a particular answer to each question.

Each of the four IMI scales (interest/enjoyment, effort/importance, perceived competence, and pressure/tension) was analyzed with two mixed-measures analyses of variance (ANOVA):

- a mixed-measures ANOVA with game type (four levels corresponding to four games) as the within-subject variable and co-player (two levels: friend/relative or therapist) as the between-subjects variable. This was done with data from all 29 participants.
- a mixed-measures ANOVA with game type as the within-subjects variable and favorite game (two levels: competition or cooperation) as the between-subjects variable. The favorite game was obtained from the overall experience questionnaire, and both cooperative games were merged into one group. As only a small number of participants (*N =* 5, as described later) chose the single-player game as their favorite, these five participants were not included in this ANOVA. Thus, the second ANOVA was done with data from 24 participants (those who did not pick single-player as their favorite).

Main effects of game type and co-player as well as interaction effects between game type and co-player were obtained from the first ANOVA while main effects of favorite game and interaction effects between game type and favorite game were obtained from the second ANOVA. The threshold for significance was set at *p =* 0.05. Effect size is reported as partial eta squared. The Greenhouse-Geisser correction was used for violations of sphericity. We acknowledge that this is not an ideal analysis and that an alternative would have been to, e.g., have a single ANOVA with three or even four levels for the favorite game variable, but this would have resulted in a very small number of subjects per group. The approach of separating participants into those who prefer competition and those who prefer cooperation was used in our previous study with good results [26] and is thus repeated here.

For the personality questionnaire, Spearman correlation coefficients (r_s_) were first calculated between each personality score and IMI responses to different games. We then used t-tests to check for personality differences between people whose favorite game (obtained from the overall experience questionnaire) was the competitive one and people whose favorite game was one of the two cooperative games. This was done only for impaired participants who exercised together with an unimpaired friend/relative. Participants who exercised with a therapist did not fill out the personality questionnaire.

#### Subjective experience – Unimpaired participants

The overall experience questionnaire was analyzed using descriptive statistics to determine how many participants gave a particular answer to each question. Each of the four IMI scales was analyzed using a mixed-measures analysis of variance with one within-subject variable (game type - three levels corresponding to three games, as unimpaired participants did not play the single-player game) and one between-subjects variable: favorite game (two levels: competition or cooperation). The entire analysis was done only for the 19 friends/relatives; the occupational therapist was not included.

#### Exercise intensity

Exercise intensity was analyzed only for impaired participants. RMS and mean absolute values of hand velocity were analyzed using two mixed-measures ANOVAs. The process was identical to that used to analyze results of the IMI in the “Subjective experience – Impaired participants” section.

## Results

### Subjective experience – Impaired participants

#### Overall experience

**Fig. 5:**
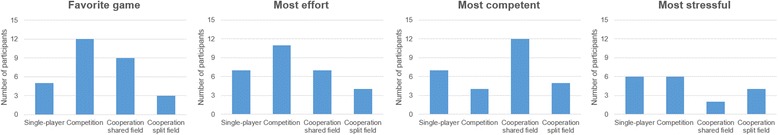
Results of the overall experience questionnaire for all impaired participants. Presented as numbers of participants that chose a particular game in response to the questions “What was your favorite game?”, “Which game did you put the most effort into?”, “What game did you feel the most competent at?”, and “Which game was the most stressful?”

Results of the overall experience questionnaire, which was presented after all four games, are shown in Table 1 for different subgroups of impaired participants. Furthermore, the main results of the questionnaire are shown in Fig. 5. To briefly summarize: The most frequently chosen favorite game was competition, and it was also the most frequently chosen as the one requiring the most effort. Cooperation with shared field was the most frequently chosen as resulting in the highest competence level. The single-player game was the most frequently chosen as least favorite, least effort and least competent.

**Table 1:** Results of the overall experience questionnaire for impaired participants only

	Single-player	Competition	Cooperation shared field	Cooperation split field
Favorite game	5	12	9	3
played with friend/relative (*N =* 19)	1	10	6	2
played with therapist (*N =* 10)	4	2	3	1
Least favorite game	11	3	4	11
played with friend/relative (*N =* 19)	8	3	2	6
played with therapist (*N =* 10)	3	0	2	5
Most effort	7	11	7	4
played with friend/relative (*N =* 19)	4	10	3	2
played with therapist (*N =* 10)	3	1	4	2
Least effort	10	2	3	9
played with friend/relative (*N =* 15)	6	2	1	6
played with therapist (*N =* 9)	4	0	2	3
Most competent	7	4	12	5
played with friend/relative (*N =* 18)	5	4	6	3
played with therapist (*N =* 10)	2	0	6	2
Least competent	10	9	0	6
played with friend/relative (*N =* 16)	5	6	0	5
played with therapist (*N =* 9)	5	3	0	1
Most stressful	6	6	2	4
played with friend/relative (*N =* 15)	5	6	1	3
played with therapist (*N =* 3)	1	0	1	1
Least stressful	4	2	6	3
played with friend/relative (*N =* 12)	2	2	6	2
played with therapist (*N =* 3)	2	0	0	1

Presented separately for participants who played with an unimpaired friend/relative and for participants who played with an occupational therapist. As some participants did not answer all the questions, the number of answers is given for each questionnaire item

Regarding participant numbers: several impaired participants declined to answer some questions on the grounds that they did not feel particularly stressed or incompetent in any game. For example, 7 of 10 participants who played with a therapist did not answer the stress questions, as all 7 stated that none of the games were stressful.

#### Experience in each game

**Fig. 6:**
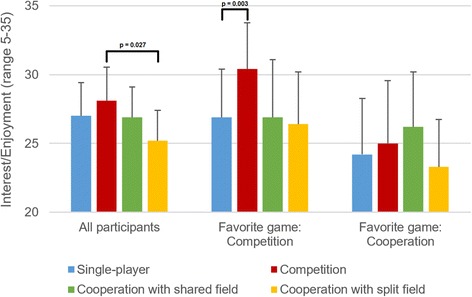
Self-reported interest/enjoyment in different games for all impaired participants (*N =* 29), for participants whose favorite game was the competitive one (*N =* 12), and for participants whose favorite game was one of the two cooperative ones (*N =* 12). Presented as means and 95% confidence intervals

**Fig. 7:**
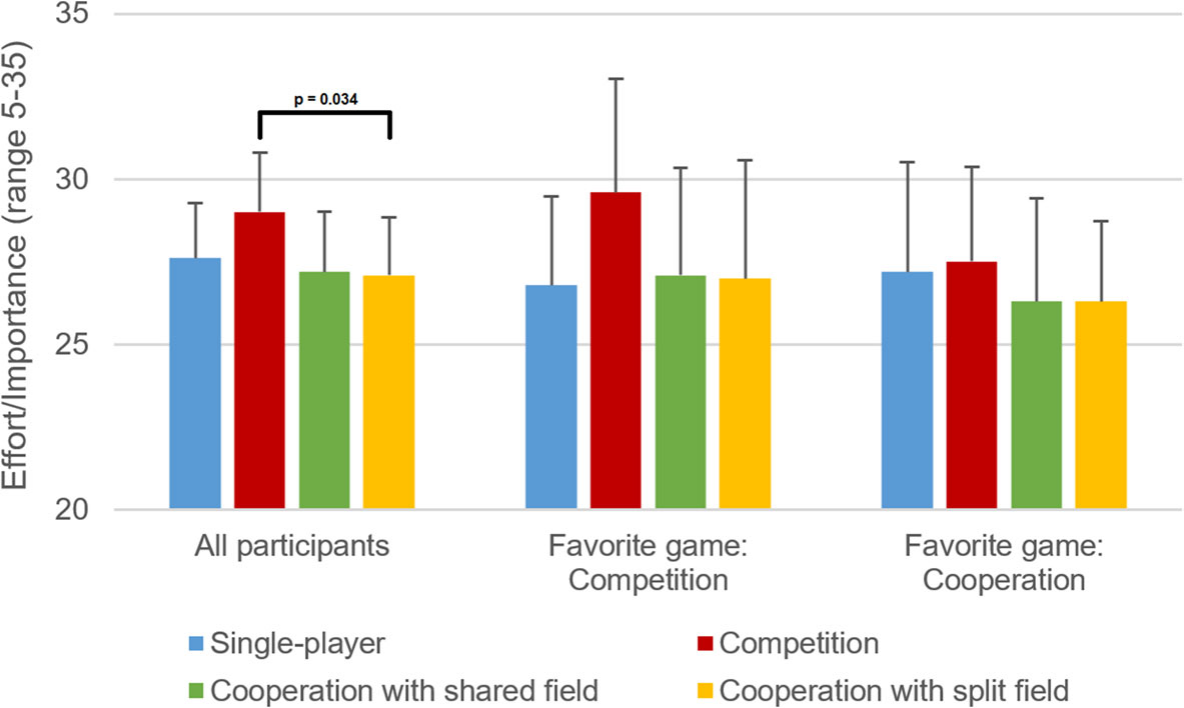
Self-reported effort/importance in different games for all impaired participants (*N =* 29), for participants whose favorite game was the competitive one (*N =* 12), and for participants whose favorite game was one of the two cooperative ones (*N =* 12). Presented as means and 95% confidence intervals

Results of the Intrinsic Motivation Inventory are presented in Table 2 for impaired participants. These results were analyzed using mixed-measures ANOVA, and the findings of that analysis are presented in Table 3. Effects of game type and favorite game on interest/enjoyment and effort/importance are also illustrated graphically in Figs. 6 and 7.

**Table 2:** Results of the Intrinsic Motivation Inventory for impaired participants

		Single-player	Competition	Cooperation shared field	Cooperation split field
Interest/ Enjoyment	Overall	26.9 ± 6.3	28.0 ± 6.3	26.9 ± 5.8	25.2 ± 5.7
	Favorite single-player	34.2 ± 0.8	30.2 ± 3.3	28.4 ± 2.4	27.2 ± 5.8
	Favorite competitive	26.6 ± 5.2	30.4 ± 5.3	26.9 ± 6.6	26.3 ± 5.7
	Favorite cooperative	24.2 ± 6.4	24.8 ± 7.0	26.2 ± 6.3	23.3 ± 5.4
	With friend/relative	25.1 ± 6.4	27.0 ± 7.0	25.8 ± 6.5	24.4 ± 5.9
	With therapist	30.4 ± 4.4	30.0 ± 4.2	28.9 ± 3.8	26.7 ± 5.0
Effort/ Importance	Overall	27.2 ± 4.7	29.3 ± 4.3	27.2 ± 4.8	27.0 ± 4.4
	Favorite single-player	30.2 ± 0.4	31.4 ± 2.3	29.8 ± 4.0	29.4 ± 3.6
	Favorite competitive	26.2 ± 4.7	30.3 ± 4.2	27.1 ± 5.1	26.8 ± 5.3
	Favorite cooperative	27.0 ± 5.4	27.5 ± 4.5	26.3 ± 4.9	26.3 ± 3.7
	With friend/relative	27.0 ± 5.2	29.7 ± 4.4	27.0 ± 5.0	27.6 ± 4.1
	With therapist	27.6 ± 4.0	28.6 ± 4.1	27.7 ± 4.7	26.0 ± 5.1
Competence	Overall	22.7 ± 6.6	23.6 ± 5.9	23.9 ± 6.0	22.7 ± 6.0
	Favorite single-player	27.0 ± 7.4	22.6 ± 5.6	27.0 ± 4.1	26.0 ± 4.3
	Favorite competitive	22.8 ± 7.5	24.9 ± 6.2	23.0 ± 7.4	22.4 ± 7.8
	Favorite cooperative	20.8 ± 4.8	22.8 ± 6.0	23.4 ± 4.9	21.7 ± 4.2
	With friend/relative	22.5 ± 6.9	24.6 ± 6.6	23.4 ± 6.4	22.8 ± 6.6
	With therapist	23.1 ± 6.5	21.7 ± 3.9	24.7 ± 5.2	22.5 ± 5.0
Pressure/ Tension	Overall	13.1 ± 6.4	13.1 ± 6.7	13.3 ± 7.4	13.6 ± 7.5
	Favorite single-player	9.0 ± 2.9	11.0 ± 5.9	11.2 ± 8.1	10.0 ± 4.1
	Favorite competitive	14.2 ± 7.3	14.1 ± 7.0	14.8 ± 7.7	15.3 ± 8.3
	Favorite cooperative	13.7 ± 6.1	12.9 ± 7.0	12.8 ± 7.1	13.4 ± 7.6
	With friend/relative	13.8 ± 7.1	13.6 ± 7.5	13.7 ± 7.9	14.6 ± 8.1
	With therapist	11.6 ± 4.8	12.1 ± 4.9	12.6 ± 6.6	11.8 ± 5.9

Presented as mean ± SD for all participants (*N =* 29) and for different subgroups

**Table 3:** Results of the mixed-measures ANOVA done on the Intrinsic Motivation Inventory for impaired participants

		p	partial η^2^
Interest/Enjoyment	Main effect of game type	**0.013**	**0.13**
	Main effect of co-player	0.099	0.098
	Main effect of favorite game	0.20	0.074
	Interaction of game type * favorite game	**0.046**	**0.12**
	Interaction of game type * co-player	0.36	0.039
Effort/Importance	Main effect of game type	**0.011**	**0.13**
	Main effect of co-player	0.83	0.002
	Main effect of favorite game	0.66	0.009
	Interaction of game type * favorite game	0.13	0.083
	Interaction of game type * co-player	0.28	0.046
Perceived competence	Main effect of game type	0.27	0.047
	Main effect of co-player	0.88	0.001
	Main effect of favorite game	0.65	0.010
	Interaction of game type * favorite game	0.29	0.055
	Interaction of game type * co-player	0.052	0.096
Pressure/Tension	Main effect of game type	0.92	0.006
	Main effect of co-player	0.46	0.02
	Main effect of favorite game	0.62	0.012
	Interaction of game type * favorite game	0.70	0.016
	Interaction of game type * co-player	0.68	0.015

Presented as *p-*values and partial η^2^ for different main and interaction effects, with significant results bolded

As seen in Table 3, there were three significant effects, and post-hoc Sidak tests were performed for all three. For the main effect of game type on interest/enjoyment, post-hoc tests found that the competitive game was more enjoyable than the cooperative one with the split field (*p =* 0.027), but no other significant differences. For the main effect of game type on effort/importance, post-hoc tests found that the competitive game resulted in higher effort/importance than the cooperative game with split field (*p =* 0.034), but no other significant differences.

For the interaction effect of game type * favorite game on interest/enjoyment, participants who chose the competitive game as their favorite had a higher interest/enjoyment in the competitive game than in the single-player game (*p =* 0.003), but not significantly higher than in the cooperative game with shared field (*p =* 0.078) or in the cooperative game with split field (*p =* 0.055). Participants who chose a cooperative game as their favorite exhibited the highest interest/enjoyment in the cooperative game with shared field, but the differences between the four games were not significant.

#### Personality

Seventeen impaired participants filled out the personality questionnaire, of which 10 chose the competitive game as their favorite, 6 chose one of the two cooperative games as their favorite, and one chose the single-player game as their favorite. No personality score showed significant differences between participants who favored competition and those who favored cooperation.

For each game, Spearman correlation coefficients were correlated between personality scores and the four IMI scales. This resulted in a total of 96 calculated coefficients (4 games × 6 personality × 4 IMI), though only significant results are reported to save space. Interest/enjoyment was correlated with agreeableness in the single-player game (r_s_ = 0.49, *p =* 0.044) and with competitiveness in the cooperative game with split field (r_s_ = −0.67, *p =* 0.003). Effort/importance was correlated with agreeableness in the single-player game (r_s_ = 0.52, *p =* 0.031), in the cooperative game with shared field (r_s_ = 0.67, *p =* 0.003), and in the cooperative game with split field (r_s_ = 0.62, *p =* 0.007). It was also correlated with intellect/imagination in the single-player game (r_s_ = 0.60, *p =* 0.01), in the cooperative game with shared field (ρ = 0.64, *p =* 0.006), and in the cooperative game with split field (r_s_ = 0.68, *p =* 0.003). Finally, perceived competence was correlated with competitiveness in the cooperative game with split field (r_s_ = −0.52, *p =* 0.034).

### Subjective experience – Unimpaired participants

Results of the overall experience questionnaire are shown for unimpaired friends/relatives in Table 4. The competitive game was the favorite of the majority of unimpaired participants (11 of 19), resulted in the highest self-reported effort, and was the most stressful. Additionally, cooperation with the split field was the least positively received game. Finally, in 12 of 19 pairs, both the impaired and unimpaired participant had the same favorite game.

**Table 4:** Results of the overall experience questionnaire for unimpaired friends/relatives only

	Competition	Cooperation shared field	Cooperation split field
Favorite	11	5	3
Least favorite	3	4	10
Most effort	10	5	2
Least effort	4	4	8
Most competent	5	6	5
Least competent	7	3	5
Most stressful	8	2	5
Least stressful	3	6	4

While there were 19 participants, not all answered all questions

Results of the Intrinsic Motivation Inventory are presented in Table 5 for unimpaired friends/relatives. These results were analyzed using mixed-measures ANOVA, and the findings of that analysis are presented in Table 6.

**Table 5:** Results of the Intrinsic Motivation Inventory for unimpaired participants

		Competition	Cooperation shared field	Cooperation split field
Interest/ Enjoyment	Overall	25.7 ± 4.5	24.3 ± 5.3	23.1 ± 5.5
	Favorite competitive	25.6 ± 5.1	22.6 ± 5.2	21.9 ± 6.1
	Favorite cooperative	25.9 ± 4.1	26.7 ± 4.8	24.9 ± 4.6
Effort/ Importance	Overall	25.1 ± 5.9	25.1 ± 7.1	25.2 ± 5.6
	Favorite competitive	27.2 ± 5.0	24.2 ± 8.0	23.2 ± 6.1
	Favorite cooperative	22.6 ± 6.2	26.1 ± 6.2	27.6 ± 4.0
Competence	Overall	22.4 ± 5.2	24.1 ± 5.5	23.3 ± 6.5
	Favorite competitive	24.8 ± 5.0	26.3 ± 3.6	25.1 ± 5.6
	Favorite cooperative	19.6 ± 4.2	21.3 ± 6.3	21.1 ± 7.3
Pressure/ Tension	Overall	14.7 ± 6.8	14.9 ± 8.3	15.3 ± 8.2
	Favorite competitive	14.4 ± 6.7	14.5 ± 7.1	15.2 ± 7.6
	Favorite cooperative	15.0 ± 7.3	15.5 ± 10.1	15.4 ± 9.4

Presented as mean ± SD for all participants (*N =* 19) and for two subgroups: those who chose the competitive game as their favorite (*N =* 11) and those who chose one of the cooperative games as their favorite (*N =* 8)

**Table 6:** Results of the mixed-measures ANOVA done on the Intrinsic Motivation Inventory for unimpaired participants

		p	partial η^2^
Interest/Enjoyment	Main effect of game type	**0.011**	**0.31**
	Main effect of favorite game	0.25	0.10
	Interaction of game type * favorite game	**0.028**	**0.25**
Effort/Importance	Main effect of game type	0.93	0.005
	Main effect of favorite game	0.811	0.004
	Interaction of game type * favorite game	**<0.001**	**0.41**
Perceived competence	Main effect of game type	0.66	0.024
	Main effect of favorite game	**0.033**	**0.27**
	Interaction of game type * favorite game	0.86	0.008
Pressure/Tension	Main effect of game type	0.91	0.004
	Main effect of favorite game	0.82	0.004
	Interaction of game type * favorite game	0.85	0.007

Presented as p-values and partial η^2^ for different main and interaction effects, with significant results bolded

As seen in Table 6, there were four significant effects, and post-hoc Sidak tests were performed for all four. For the main effect of game type on interest/enjoyment, post-hoc tests found that the competitive game was more enjoyable than the cooperative one with the split field (*p =* 0.033), but no other significant differences. For the main effect of favorite game on perceived competence, participants who chose the competitive game as their favorite had a significantly higher overall level of competence (*p =* 0.033).

For the interaction effect of game type * favorite game on interest/enjoyment, participants who chose the competitive game as their favorite had a higher interest/enjoyment in the competitive game than in the cooperative game with shared field (*p =* 0.045) and in the cooperative game with split field (*p =* 0.021). Participants who chose a cooperative game as their favorite exhibited no significant differences between games. For the interaction effect of game type * favorite game on effort/importance, participants who chose the competitive game as their favorite had a higher effort/importance in the competitive game than in the cooperative game with split field (*p =* 0.036). Participants who chose a cooperative game as their favorite had a higher effort/importance in the cooperative game with shared field than in the competitive game (*p =* 0.043).

Finally, no personality score showed significant differences between participants who favored competition (*N =* 11) and those who favored cooperation (*N =* 8).

### Exercise intensity

**Fig. 8:**
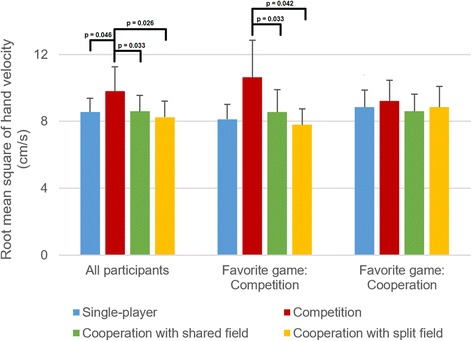
Root-mean-square values of hand velocity in different games for all impaired participants (*N =* 28), for participants whose favorite game was the competitive one (*N =* 12), and for participants whose favorite game was one of the two cooperative ones (*N =* 12). Presented as means and 95% confidence intervals

One impaired participant’s hand velocity data was lost due to a logging error, resulting in valid data for 28 participants. The RMS and mean absolute values of hand velocity are presented in Table 7. These results were analyzed using mixed-measures ANOVA, and the findings of that analysis are presented in Table 8. Effects of game type and favorite game on RMS values of hand velocity are also illustrated graphically in Fig. 8.

**Table 7:** Root-mean-square and mean absolute values of hand velocity for all four games

		Single-player	Competition	Cooperation shared field	Cooperation split field
Root mean square value (cm/s)	Overall	8.36 ± 2.09	9.96 ± 3.68	8.63 ± 2.42	8.15 ± 2.48
	Favorite single-player	8.89 ± 0.84	9.13 ± 2.28	8.76 ± 1.41	7.66 ± 1.95
	Favorite competitive	7.89 ± 2.03	10.90 ± 4.79	8.51 ± 2.95	7.75 ± 2.41
	Favorite cooperative	8.66 ± 2.45	9.29 ± 2.69	8.71 ± 2.24	8.67 ± 2.73
	With friend/relative	8.51 ± 2.35	9.83 ± 2.51	8.61 ± 2.33	7.94 ± 2.67
	With therapist	8.05 ± 1.47	10.24 ± 5.61	8.67 ± 2.74	8.66 ± 2.02
Mean absolute value (cm/s)	Overall	5.14 ± 1.42	6.26 ± 2.60	5.43 ± 1.76	4.80 ± 1.70
	Favorite single-player	5.69 ± 0.95	6.11 ± 1.99	5.93 ± 1.27	4.88 ± 1.47
	Favorite competitive	4.77 ± 1.37	6.84 ± 3.36	5.31 ± 2.14	4.37 ± 1.50
	Favorite cooperative	5.33 ± 1.59	5.72 ± 1.87	5.39 ± 1.57	5.22 ± 1.95
	With friend/relative	5.22 ± 1.59	6.11 ± 1.78	5.38 ± 1.73	4.64 ± 1.85
	With therapist	4.98 ± 1.02	6.58 ± 3.94	5.55 ± 1.92	5.19 ± 1.30

Presented as mean ± SD for all impaired participants (*N =* 28) and for different subgroups

**Table 8:** Results of the mixed-measures ANOVA done on root-mean-square and mean absolute values of hand velocity

		p	partial η^2^
Root mean square value	Main effect of game type	**0.003**	**0.21**
	Main effect of co-player	0.79	0.003
	Main effect of favorite game	0.95	0.000
	Interaction of game type * favorite game	**0.048**	**0.13**
	Interaction of game type * co-player	0.53	0.025
Mean absolute value	Main effect of game type	**0.002**	**0.23**
	Main effect of co-player	0.65	0.008
	Main effect of favorite game	0.90	0.001
	Interaction of game type * favorite game	**0.043**	**0.13**
	Interaction of game type * co-player	0.55	0.024

Presented as *p-*values and partial η^2^ for different main and interaction effects, with significant results bolded

As seen in Table 8, there were four significant effects, and post-hoc Sidak tests were performed for all four. For the main effect of game type on the RMS value of hand velocity, post-hoc tests found that the competitive game resulted in a higher RMS value than the single-player game (*p =* 0.046), the cooperative game with shared field (*p =* 0.033) and the cooperative game with split field (*p =* 0.026). For the main effect of game type on the mean absolute value of hand velocity, post-hoc tests found that the competitive game resulted in a higher mean absolute value than the single-player game (*p =* 0.048) and the cooperative game with split field (*p =* 0.012), but not the cooperative game with shared field (*p =* 0.051).

For the interaction effect of game type * favorite game on the RMS value of hand velocity, participants who chose the competitive game as their favorite had a higher RMS value in the competitive game than in the cooperative game with shared field (*p =* 0.042) and the cooperative game with split field (*p =* 0.033), but not in the single-player game (*p =* 0.093). For participants who chose a cooperative game as their favorite, there were no significant differences in post-hoc tests. For the interaction effect of game type * favorite game on the mean absolute value of hand velocity, participants who chose the competitive game as their favorite had a higher mean absolute value in the competitive game than in the cooperative game with split field (*p =* 0.030), but not in the single-player game (*p =* 0.12) or the cooperative game with shared field (*p =* 0.063). For participants who chose a cooperative game as their favorite, there were no significant differences in post-hoc tests.

## Discussion

### Game preferences

As noted in our previous study with healthy subjects [26], both competition and cooperation can increase patient motivation. Of 29 impaired participants, only 5 chose exercising alone as their favorite. Twelve impaired participants chose competition as their favorite game while twelve chose one of the two cooperative games, suggesting that (at least for this game type) there is no interpersonal interaction type that would be preferred by the majority of participants. Interestingly, only 3 of 29 impaired participants chose the competitive game as their least favorite, and only 2 of 15 chose it as the most stressful, suggesting that **competing with an unimpaired person was not considered unpleasant**. For comparison, in our previous study [26], 23 of 30 healthy participants chose the competitive game as the most stressful.

However, the importance of the co-player must be emphasized: **impaired participants who exercised with a friend or relative were much more likely to favor competition than those who exercised with a therapist**. Similarly, impaired participants who exercised with a friend or relative were less likely to prefer exercising alone than those who exercised with a therapist. This finding may be confounded by the fact that time since injury was different in the two groups, but we nonetheless believe that exercising together with a friend or relative in a familiar setting is more motivating than exercising with a therapist at the rehabilitation clinic.

Finally, selecting the favorite game has one weakness: as the strength of the preference was not measured by the overall experience questionnaire, it is possible that some participants had no real preference and simply selected an option at random. Based on participant interviews and qualitative experimenter observations, we believe that most participants did have a preference (for example, a few declared that everything but competing was boring). Nonetheless, future studies should use an overall experience questionnaire that also asks about the strength of the preference. For example, our recent study of computer games asked participants to compare two similar games using multiple questions, and each question had multiple possible answers ranging from “no difference” to “large difference” [40].

### Motivation and exercise intensity

The competitive game resulted in the highest overall interest/enjoyment and exercise intensity. Post-hoc tests found that RMS and mean absolute values of hand velocity were higher in the competitive game than in all three other games, though this was primarily true for people who picked the competitive game as their favorite. This has an important implication for rehabilitation: **participants who like competitive rehabilitation games will exercise significantly more intensely in such games**. The finding is admittedly not surprising, as increased exercise intensity due to competition was already demonstrated in related fields such as weight loss [20]. Nonetheless, this is the first time that such increased exercise intensity was demonstrated in motor rehabilitation using impaired participants.

People who chose competitive games as their favorite, however, have the same enjoyment, self-reported effort, and exercise intensity in cooperative games as in the single-player game (Figs. 6, 7 and 8). Thus, people who enjoy competition more than exercising alone do not necessarily enjoy cooperation more than exercising alone, and do not exercise more intensely when cooperating with others than when exercising alone. People who chose a cooperative game as their favorite, on the other hand, still appear to enjoy competition (Fig. 6), but do not have higher exercise intensity in either competitive or cooperative games than in the single-player game.

Finally, though both we [26] and others [11] had raised the possibility of competition being considered stressful and unpleasant, results of the IMI suggest that this is not very problematic. Pressure/tension was approximately equal in all four games, with mean values around 13 on a scale of 5 to 35 (Table 2). **Therefore**, **neither competition nor cooperation between patients and their unimpaired friends or relatives is significantly more stressful than exercising alone**. In contrast, our 2014 study [26] used the same questionnaire and found a mean pressure/tension of 18.1 during competitive exercises performed by pairs of unimpaired participants (who were not always friends or relatives), indicating that competition can be more stressful in such participant pairs.

### Potential long-term benefits and our next steps

Based on our results, we posit that competitive games played by patients together with unimpaired friends and relatives have the highest potential for motor rehabilitation, as they increase both motivation (Fig. 6) and exercise intensity (Fig. 8). Since motivation [13, 14] and exercise intensity [41, 42] are positively correlated with long-term rehabilitation outcome, competitive rehabilitation games could, in the long term, lead to improved arm function and increased quality of life. Cooperative rehabilitation games do not necessarily increase exercise intensity, but could still result in long-term benefits by increasing motivation.

We acknowledge, however, that our results were obtained only for a single brief session, and that the increased motivation and exercise intensity might not persist over a longer session or over multiple sessions. The brief duration of each game in our study was selected so that participants could evaluate multiple games within a single session without becoming tired, similarly to our previous study [26]. However, this is not optimal, and our next steps will focus on evaluating competitive rehabilitation exercises over multiple longer sessions.

Before beginning a multisession study, we will first augment competitive exercises with difficulty adaptation algorithms that intelligently tailor the exercise difficulty to both participants. These will ensure that the patient achieves optimal exercise intensity while the unimpaired person is not bored by the exercise. They will be based on previously proposed adaptation algorithms for competitive games [28, 43, 44] as well as adaptation algorithms for rehabilitation exercises performed by a single patient [8, 45].

Once the difficulty adaptation algorithms have been implemented and tested, we will test the adaptive competitive exercises over 3–4 sessions. While this is not a long enough time period to study improvements in functional arm ability, it will allow us to determine whether increased motivation and exercise intensity persist over a longer period. For example, potential benefits of competition may fade over multiple sessions as participants become used to the exercise and the novelty wears off. Alternatively, competition may increase aggression and frustration (as seen in competitive games for weight loss [20]), making participants unwilling to exercise again. These effects should be measurable over 3–4 sessions, allowing us to gauge “medium-term” effects of competitive exercises before launching a full clinical trial.

Admittedly, the observed increases in enjoyment and motivation were not large. For example, the RMS value of hand velocity was 20% higher in the competitive game than in the single-player game. Similarly, for participants who chose the competitive game as their favorite, interest/enjoyment was about 15% higher in the competitive game than in the single-player one. Even if these increases persist over time, they may not be sufficient to improve long-term rehabilitation outcome. However, this issue is not specific to competitive exercises; while motivation and exercise intensity are known to be correlated with motor rehabilitation outcome, researchers have not yet identified minimal clinically important differences in either motivation or exercise intensity. Further research into the relationship between motivation, exercise intensity and rehabilitation outcome is thus necessary to allow better evaluation of motivational rehabilitation interventions.

### Other game designs

Our results were obtained using multiple variants of the same game (Pong), and may not apply to other competitive or cooperative game designs. For example, it is perhaps not surprising that competition had the ‘best’ results in our study, as even cooperative variants of Pong include some competition (a computer opponent). This may explain why participants who favored competition had an overall higher interest/enjoyment on the IMI than participants who favored cooperation. Greater benefits of cooperation, however, could potentially be achieved with a different game design.

#### Improved cooperative rehabilitation games

There are several game designs that could be more fun when cooperating than when competing. For example, a popular virtual environment for rehabilitation is the “virtual kitchen”, where patients cook dishes by picking up different ingredients and placing them into a pot or pan (e.g. Guidali et al. [46]). Such a virtual environment could be reasonably converted into a cooperative game, where two people prepare meals together. Another option would be a game where two participants hold a large object and jointly move it around, avoiding obstacles. Such a game was previously proposed for rehabilitation [27], and is currently being evaluated with unimpaired subjects [47].

Such cooperative games should ensure that the unimpaired participant does not reduce the exercise intensity for the impaired participant. For example, in our cooperative game with split field, the incoming balls are evenly split between both participants, and the impaired participant can thus be idle half the time. The impaired participant could also remain idle in the cooperative game with shared field and rely on the unimpaired ‘back-up’ to deflect the incoming balls; however, all of our impaired participants tried to actively intercept the balls themselves.

We also believe that cooperative games would benefit from haptic interaction between the two participants. For example, Ganesh et al. [48] demonstrated that motor learning can be accelerated by haptically coupling two people with a spring-damper system and suggested that this could be beneficial for motor rehabilitation. Our previous research showed the technical feasibility of therapists teaching arm motions to patients using two coupled arm rehabilitation robots [49]. Thus, while competitive games may be better for motivation and exercise intensity, we believe that cooperative games would be beneficial from the perspective of motor learning.

Such haptic cooperative games could take advantage of fundamental research in human-human haptic interaction, which has shown that dyads perform many tasks better than individuals despite imperfect coordination and differences in skill [50, 51]. Additionally, objective measures of haptic cooperation [52] could be used to determine whether both participants are contributing to the task or whether the difficulty needs to be adapted to balance the contributions of both participants. Similar metrics of interlimb coordination have been used in bimanual rehabilitation to ensure that hemiparetic patients use both arms to actively contribute to the task [53].

#### Games where participants should exercise alone

Five participants stated that they preferred exercising alone to both competition and cooperation. Due to the small sample size, their experience was not analyzed in detail. However, in a follow-up discussion, two of these participants stated that they found the therapist co-player distracting: by reminding them to focus on factors such as motion quality, the therapist kept them from focusing on game-like aspects. Based on these interviews and our experience with other rehabilitation games, we believe that both competition and cooperation are unsuitable for games where a strong cognitive focus is required.

### Experience of friends and relatives

Previous studies expressed concern that unimpaired friends and relatives may feel uncomfortable competing with patients [11]. However, our results show that this was generally not a problem: most unimpaired participants chose the competitive game as their favorite (Table 4), and their mean pressure/tension values were similar for all games (Table 5). The IMI analysis does show that unimpaired participants who favored the competitive game put higher effort into that game while participants who favored cooperative games put higher effort into those games. Therefore, unimpaired people who enjoy competition are likely to provide more of a challenge for the patient, and unimpaired people who enjoy cooperation are likely to provide more assistance to the patient.

### Measuring the effects of interpersonal rehabilitation games

Our study measured motivation using questionnaires and exercise intensity using sensors built into the BiMeo. Since both motivation and exercise intensity are correlated with rehabilitation outcome, measuring these two quantities over a small number of sessions is a necessary step prior to conducting long-term clinical trials. However, additional measures of motivation, exercise intensity and exercise quality should be considered for future studies.

#### Motivation

We previously noted that the IMI is poor at identifying differences between multiple similar exercises [26]. We used it again in this study since it the most popular validated motivation questionnaire in rehabilitation. However, for future research, we recommend using a shorter, simpler questionnaire. For example, the standard IMI includes negative items such as “I couldn’t play the game very well” that the subject should agree or disagree with, but many participants get confused about what disagreeing with such a negative statement actually implies. We are currently testing a modified IMI that contains no negative items and will hopefully be more suitable for patients with neurological injuries.

Furthermore, motivation does not need to be measured only with questionnaires. Our previous research on cognitive rehabilitation exercises indicates that motivated participants will exercise for longer periods of time [54], so we could measure motivation indirectly by letting participants exercise as long as they want, then seeing if they exercise longer in a certain game. This would serve as a more objective measure compared to questionnaires, and would also let us better estimate whether a certain game would make patients exercise more often or for longer periods of time.

#### Exercise intensity and motion quality

We measured exercise intensity via the limb velocity measured by the BiMeo’s inertial sensors. While this is a commonly used metric of exercise intensity in arm exercise [38, 39], it is still somewhat nonstandard and difficult to compare between different motion types. Therefore, future studies could consider alternative measures of exercise intensity, such as heart rate, muscle activation (measured via electromyography), or oxygen consumption.

Finally, we should examine whether the motions performed by patients in competitive or cooperative exercises are actually useful for rehabilitation. In a few of our participants, for example, high exercise intensity was accompanied by an increase in compensatory motions, where the participant moved their torso instead of the arm in order to play the game. Since such compensatory motions do not help improve limb motor function [55, 56], future studies should try to quantify not only the intensity, but also the quality of the performed motions.

## Conclusions

Our study demonstrated that competitive rehabilitation games played by a patient and unimpaired person have the potential to improve both motivation and exercise intensity, though only in patients who enjoy competition. Cooperative games also increase motivation, but do not increase exercise intensity. Furthermore, patients who exercise together with friends or relatives are much more likely to enjoy competition than those who exercise together with therapists. Neither competition nor cooperation with an unimpaired person are significantly more stressful for patients than exercising alone.

As competitive games increase both motivation and exercise intensity, we believe that they have high potential to lead to functional improvement and increased quality of life for patients. However, as the observed increases in motivation and exercise intensity were evaluated over only a single brief session, the crucial next research step is to augment competitive games with dynamic difficulty adaptation algorithms, then test them in multisession studies to determine whether the increases in motivation and exercise intensity are maintained over multiple sessions and whether they are large enough to meaningfully impact rehabilitation outcome.

Furthermore, we acknowledge that competitive rehabilitation games are not suitable for everyone. While cooperative games do not increase exercise intensity, they nonetheless do increase motivation, and we believe that they could be improved further with design elements that require participants to coordinate their motions with each other in order to achieve a task. Such games could include elements of haptic interaction and would have the potential to improve motor learning in rehabilitation, usefully complementing competitive games.

## Additional file

Additional file 1: Raw data (questionnaire results and hand velocity) for all participants. (XLSX 24 kb)

## Abbreviations

ANOVA: Analysis of variance
IMI: Intrinsic Motivation Inventory
IMU: Inertial measurement unit
IPIP: International Personality Item Pool
RMS: Root mean square
SD: Standard deviation
